# Tartar and Its Removal from the Teeth

**Published:** 1883-09

**Authors:** Theodore F. Chupein

**Affiliations:** Philadelphia.


ARTICLE III.

### TARTAR, AND ITS REMOVAL FROM THE TEETH.

---

BY THEODORE F. CHUPEIN, D. D. S., PHILADELPHIA.

---

This is a subject which should claim more attention from the dentist than it generally does.

As a general rule the text-books treat the subject with only a passing notice. The last edition of *Taft's Operative Dentistry* devotes but a few pages to the entire subject, while the instruments illustrated therein for its removal are clumsy, old-fashioned, and ill adapted to the operation.

Many valuable teeth are lost by becoming coated with this substance, which, not being removed while in the soft or chalky state, assumes a hard, sometimes a flinty, consistency, gradually pushing away the gums before it, causing them to bleed at the slightest touch, giving rise to fetid breath, and eventually causing a recession of the gums, an absorption of the sockets, thereby leaving the teeth with no firm support, when they loosen, and become in this state such a source of annoyance, and sometimes of pain, as to demand their removal. Even before the case has assumed such proportion as is related above, a careful examination will show, high above the free margins of the gums, rings or nodules of hard tartar, and the teeth slightly loose. If the case be taken in hand at this point there may be a chance to restore it to a normal condition by removing *all the tartar;* but if it be permitted to encroach until it reaches the alveolar border or beyond the reach of the instruments used for its removal it becomes almost impossible of removal, or so painful that the patients can or will submit to its entire removal: while in those who do submit to the operation the flow of blood from the irritated gums is so excessive as to leave the operator in doubt as to whether the work has been thoroughly performed. We have seen cases where the teeth have been so encrusted that they have actually been hidden from view by excess of the deposit, and to remove the tartar when it has been allowed to increase to this extent would be equal to the extraction of the teeth; for bound together in this condition the tartar alone held them in the mouth, and if it were removed the teeth would fall out from the lack of support.

It is not our attention, in this essay, to go into an explanation of the constituents of tartar, its analysis, or the proportions of animal and mineral matter it contains, or of such general facts in relation to it as are well known to both the student and practitioner of dentistry. We purpose merely to offer some thoughts on the subject, as well as some practical suggestions for the cleansing of the teeth that have become coated with this substance.

Tartar is an elimination of the saliva, and attaches itself —after becoming precipitated—to the teeth nearest the salivary ducts, from which the saliva flows into the mouth. Thus we find it most excessive and attached to the first and second upper molars, on each side, on their buccal sufaces, and to the lingual surface of the lower incisors and cuspids, these teeth being most contiguous to the superior and inferior salivary ducts.

If the food were vigorously chewed it is doubtful if tartar would collect on the teeth at all. In evidence of this it is seldom found on the teeth of habitual tobacco chewers or on the teeth of such domestic animals as horses, sheep, goats, cows or other ruminantia, while this assumption is still farther corroborated in the evidence we all have had of patients, who, having an aching tooth on one side of the mouth, chew their food entirely on the other side to avoid pain, when we find that the teeth on the side which has been inactive become thickly coated with tartar, while the other side, where the teeth have been vigorously used, is almost or entirely free from any deposit.

It is not always, however, that these teeth which are in the immediate vicinity of the salivary ducts become coated with tartar. We find it at times showing a peculiar fancy for one particular tooth remote from these points; sometimes a central incisor or lateral of the upper jaw being a victim to the overweening affection of this insidious destroyer, while the other teeth where we might expect it are comparatively free of this deposit, and thus many a beautiful tooth, " without spot or blemish " of decay, has to be removed from looseness caused by the tartar having insinuated itself so far on to the root as to produce absorption of the socket, destruction of the investing membrane, the recession of the gum, and, as a consequence of these, the loss of the tooth.

We find an absence of tartar in certain forms of dyspepsia; the frequent acid eructations of this disease seeming to act on the mineral constituents of this substance, thereby keeping the teeth free from any excessive deposit. In pregnant women, too, there is sometime an absence of any large accumulation of tartar on the teeth. This may be attributed to a like cause: for there seems to be inseparable to this state so strong an acid condition of the system that one lady is wont to speak of a friend or acquaintance in this condition as being "off on a vinegar voyage."

Physiologist have not as yet determined the use of tartar.

Tartar takes its name from its resemblance to an incrustation found on the inside of wine casks.

Tartar is recognized under three colors—black, green and yellow. Black tartar is principally found in the mouths of smokers, and probably derives its color from the stain eliminated by the nicotine in the smoke. Green tartar is supposed to be yellow tartar which has become more dense with age. Yellow tartar is most probably the second stage or formation of the first precipitate, which is found on the teeth by a cream-colored, softish deposit, easily removed with the tooth-brush. There seems to be no chemical difference in the composition of the three varieties.

We find the surface of tartar which lies next the cheek or tongue to be smoth, while that which is contiguous to the gum is rough, Additions are generally made to the rough parts, which accounts, alike, for the great disposition of the gums to bleed at the slightest touch, as well as to their gradual recession as new deposits are added.

When tartar is of a chalky consistency it may be readily removed with almost any of the small scalers made for this operation. It is only when it has become very dense, and is almost out of sight and out of reach, that its removal becomes difficult and calls for the employment of delicate instruments and much patience for thorough work. In this condition and position acids have been suggested for its solution, but we have felt timid of resorting to these, as we felt that an acid that would dissolve the tartar might dissolve the tooth.

While filling teeth to which the rubber dam had been applied over several, we have frequently noticed rings or nodules around the teeth that were entirely out of sight and not suspected, over which the dam had worked itself. It occurred to us, therefore, that it might be a good plan to apply the dam to assist in the removal of tartar. We frequently do this, and by its aid are enabled to make very thorough work of cleansing the teeth of tartar.

We seldom attempt the removal of all the tartar at one sitting. When in a chalky condition, around the lower teeth, it is our practice to remove as much of this as we can from all the surfaces, using those thin, flexible scalers that are now found at the depots to cleanse between the teeth. These pass readily through at the necks of the teeth, and with them very thorough work can be accomplised, except when the blood flows so freely as to obliterate the view. At the next sitting it is a good plan to apply the dam over six, eight or ten teeth, forcing this down on to the soft, flabby and yeilding gums, and applying ligatures to each tooth. After a time the dam becomes very adherent, and the ligatures may be removed; and when found necessary the dam may be insinuated still farther on the teeth by forcing small pieces of spunk between the teeth. In this way, and with the aid of a mirror, well-tempered, sharp and nicely-made scalers, all the tartar can de removed, and this, too, with none of the annoyance of the free flow of blood from the gums which so frequently defeats the thorough performance of this operation, and like-wise preventing the patient's poking the tongue in the way of the operator. The rubber dam is prepared by punching six, eight or ten holes, three-sixteenths of an inch apart, and in the form of a semi-circle. It is first stretched over one tooth, on the right or left side and held down by aid of a clamp, then stretched over the next tooth, and the next, until all are completely encircled. They are then each ligated, one by one, and then the clamp may be removed. After the dam becomes adherent the ligatures may be removed, one by one, and each tooth thoroughly cleaned as each ligature is removed.

It may not be generally known that tartar is frequently more easily removed by *pushing it* than by *pulling it.* A small nodule will often defy a most vigorous pull to dislodge it, while it will yield most readily and instantly to a delicate push.

Tartar sometimes shows itself by a darkened color on the gums, or indicates its presence on the tooth by a bluish line; or the tooth, on being taken between the thumb and finger, is observed to be slightly or considerably loosened. To any of these indications it is safe to apply the dam as recommended. The pushing up of small peices of spunk produces an apsorbtion of the gums, and the dam gradually follows, when pieces of tartar heretofore invisible are brought into view, and are readily and easily removed.

Green tartar, or green stain, differs from the salivary calculus by being corrosive in its action. It is not often observed on the teeth in middle or advanced life, but mostly affects the teeth of children about the tenth, twelfth or fourteenth year of age. It is most generally found on the four or six upper front teeth. By means of soft rubber or wooden wheels charged with fine pumice and driven by the dental engine, green tartar may be readily removed.

There is a condition of the gums brought on, we think, by the accumulation of tartar. We find them red, hot, soft, flabby and spongy; bleeding profusely at the slightest touch and hanging in loose, puffy folds about the teeth, to which they have no adhesion. The flow of blood from them is so excessive that it is next to impossible to do anything towards cleansing the teeth when the gums are in this condition. The tartar on these teeth is not hard or even chalky but soft and glutinous; the saliva is viscid and ropy. In such cases it is our practice to deplete the gums freely. All the loose, flabby folds between the teeth we remove. This is best and quickest and painlessly done with a pair of sharp, curved-blade, pointed scissors. These are passed, with a blade on each side of the loose fold of gum, between the teeth, and with a quick snap of the handles the congested gum is taken off. After the gums are well rinsed with cold water and the bleeding stopped we apply to all the incised places a little "iodide of zinc," taken upon a moistened camel-hair pencil, or on a swab of moistened cotton floss. This iodide of zinc should be kept in a well-corked bottle, as it rapidly absorbs moisture from the atmosphere and runs into a semi-fluid state. At the next sitting the case will be in a better condition for the removal of the soft or mucilaginous tartar that will be found around the necks of the teeth, which will be best removed, as suggested, by the aid of the rubber dam.—*Dental Office and Laboratory.*

